# The Story of the Insane from Year to Year

**Published:** 1894-10-13

**Authors:** 


### THE STORY OF THE INSANE FROM YEAR TO YEAR.

(7th Series.)

### NOTTINGHAM BOROUGH ASYLUM.

Since its opening in 1880 this asylum has more than doubled its population, and it now contains 554 patients. Last year there were 168 admissions, 68 recoveries, and 64 deaths. The recoveries stand at the creditable percentage of 42·2, and the deaths are a little above the average at 10·9. Intemperance in drink accounts for 25 of the year's admissions, but previous attacks and hereditary influence head the list, the former with 37 and the latter with 28. Influenza is put down as the cause in 5, and 30 are unknown. Not fewer than 47 of the deaths were owing to cerebral or spinal disease, and 34 of the 47 were caused by general, or more correctly, progressive paralysis of the insane. Consumption killed only 4. Mr. Powell comments on the unfavourable nature of the admissions, and states that the majority were of a most unfavourable character, and presented little hope of improvement, much less recovery. "The visitors beg to express their entire satisfaction with the conduct of the officials and staff generally." The weekly charge for maintenance is 10s. per head.

### WARWICK COUNTY ASYLUM.

An increase from 739 to 771 has to be noted. There were 209 admissions, 84 recoveries, and 69 deaths. Put into percentages in the manner usual in asylums we find the recoveries stand at 40·2, and the deaths at 9·4. Hereditary influence, previous attacks and congenital defect taken together account for 111 of the admissions, and intemperance in drink for 18. Cerebral diseases caused death in 26 instances, consumption in 12, and dysentery in one. The admissions included 23 idiots, 17 epileptics, 10 paralytics, 73 chronic cases, and 90 acute cases. Dr. Miller says this is more favourable than in former years, and he looks forward to a good recovery rate in 1894; but as some of the acute cases will prove incurable, we doubt whether he will find it higher than in 1893. The number of certified lunatics is increasing in Warwickshire, as almost everywhere else, and there is a relative as well as actual increase, for we find Dr. Miller stating that in 1881 the number of certified lunatics in Warwickshire was 802, being at the rate of 2·45 per thousand; whereas now there are 989, being at the rate of 2·76 per thousand. Warwickshire is, however, lower than the rest of England, which shows an average of 3·02 per thousand. The weekly charge to the unions is 8s. 5½d. per head.

